# ASCANIO: a backscattering X-ray multichannel spectrometer for synchrotron applications

**DOI:** 10.1107/S1600577526006843

**Published:** 2026-08-05

**Authors:** Pietro Barcella, Giacomo Ticchi, Beatrice Pedretti, Jan Garrevoet, Dennis Brückner, Giacomo Borghi, Marco Carminati, Gerald Falkenberg, Carlo Fiorini

**Affiliations:** a Dipartimento di Elettronica, Informazione e Bioingegneria (DEIB) Politecnico di Milano Milano Italy; b Istituto Nazionale di Fisica Nucleare (INFN) Sezione di Milano Milano Italy; c Deutsches Elektronen-Synchrotron (DESY) Hamburg Germany; RIKEN SPring-8 Center, Japan

**Keywords:** X-ray spectroscopy, silicon drift detectors, backscattering spectrometry

## Abstract

X-ray spectrometers with high count rate capability are becoming essential to provide higher speeds in synchrotron measurements, like in X-ray fluorescence mapping (XFM). Monolithic multichannel silicon drift detectors (SDDs) enable the creation of dense and high-performance matrices to reach this goal. In this paper we show the results of the development of ASCANIO, a 16-channel backscattering X-ray spectrometer based on SDD monolithic modules. The solid angle and count-rate uniformity in X-ray detection have been optimized thanks to a ‘tilted’ configuration of the SDD units. ASCANIO underwent preliminary testing in the laboratory, where the system achieved around −42°C with an internal pressure of 5.1 × 10^−6^ mbar. During further verification of its spectroscopic performance, the spectrometer showed resolutions as low as 134 eV FWHM at the Mn *K*α peak. After preliminary testing the full system was commissioned on beamline P06 at PETRA III (DESY). Here the effects of the tilted configuration could be observed, and the system achieved a 20 Mcps cumulative output count rate with a dead time of 20%. As a demonstration of ASCANIO’s usability, an example use case is demonstrated, where ASCANIO was used for an XFM analysis of a biological sample.

## Abstract 2

This paper presents the development and commissioning of ASCANIO, an innovative multichannel backscattering X-ray spectrometer, including extensive testing on a synchrotron beamline, with highlights of the main results achieved.

## 1. Introduction

Advanced X-ray experimental techniques, including X-ray fluorescence (XRF), micro-XRF and X-ray absorption fine structure (XAFS), have experienced increasing adoption across multiple scientific disciplines such as materials science, biology, geology and environmental research (Marguí *et al.*, 2022 ▸). Notable capabilities include minimal sample preparation requirements (Beckhoff *et al.*, 2007 ▸), simultaneous multi-element detection (Jenkins, 1999 ▸) and the determination of elemental presence, concentration and oxidation states through X-ray absorption spectroscopy experiments (Gräfe *et al.*, 2014 ▸).

While high-quality laboratory instrumentation enables accurate X-ray measurements, synchrotron radiation sources remain indispensable for obtaining specific scientific insights due to their unique characteristics. These include energy tunability, ranging from approximately 250 eV to 100 keV or higher (Mobilio *et al.*, 2015 ▸), and pulsed X-ray emission structures that facilitate time-resolved experiments for analyzing dynamic processes on timescales from picoseconds to seconds (Schoenlein *et al.*, 2017 ▸). Furthermore, the capability to focus X-ray beams to sub-micrometre dimensions is essential for high-resolution elemental mapping and detailed spatial distribution analysis within samples (Naim *et al.*, 2021 ▸).

The technological advancement of synchrotron radiation sources continues to progress, yielding increasingly brilliant X-ray beams. Numerous facilities have undergone, or are currently undergoing, upgrades to fourth-generation configurations featuring brilliance levels up towards 10^22^ photons s^−1^ (0.1% bandwidth)^−1^ mm^−2^ mrad^−2^. For example, The ESRF Extremely Brilliant Source, one of the earliest operational fourth-generation facilities worldwide, delivers 6.6 × 10^21^ photons s^−1^ (0.1% bandwidth)^−1^ mm^−2^ mrad^−2^ at 10 keV, representing an improvement of two orders of magnitude over the previous third-generation configuration (Raimondi *et al.*, 2023 ▸).

While harnessing such high brilliance undoubtedly enhances capabilities for detecting elements at sub-p.p.m. concentrations, detection systems must be redesigned and optimized to accommodate these elevated input fluxes. Without such improvements, there is a substantial risk of under-utilizing the potential of next-generation synchrotron sources.

Over the past two decades, silicon drift detectors (SDDs) (Gatti & Rehak, 1984 ▸) have emerged as the optimal solution for beamline applications within the silicon detection energy range, owing to their superior performance in terms of high count rates, high energy resolution and low electronic noise. These detectors have largely replaced Si(Li) or HPGe detectors and gas ionization chambers at synchrotron facilities.

Maximizing X-ray fluorescence photon detection in SDD-based spectrometers requires careful optimization of three critical parameters: geometric efficiency, and low- and high-energy detection capabilities. Each parameter demands specific design considerations to minimize signal loss and reduce experimental duration. Geometric efficiency represents the first fundamental consideration in spectrometer design and mainly depends on the total active detector area and the sample-to-detector working distance. For specimens exhibiting low X-ray fluorescence yield, such as biological samples or dilute environmental matrices, the deployment of large-area detectors or multi-element detector arrays becomes particularly advantageous (Rumancev *et al.*, 2020 ▸). At the same time, the sample-to-detector working distance represents a critical optimization parameter that necessitates careful balancing: while shorter working distances maximize the solid-angle coverage enhancing detection efficiency, minimum distance thresholds must be maintained to prevent mechanical interference with sample-positioning apparatus and to protect the detector entrance window from damage during experimental operations.

Beyond maximizing the solid angle subtended by the detector, the entrance window itself significantly influences detection performance in the low-energy regime below 5 keV. Traditional 8 µm thick beryllium windows provide transmission coefficients at 1.5 keV ranging from 60% to 73%, with variation depending on manufacturing tolerances (Henke *et al.*, 1993 ▸). Advanced materials offer substantial improvements in this critical energy region: for instance, boron carbide (B_4_C) windows achieve 77% transmission at 2.1 µm thickness (Redus *et al.*, 2022 ▸), delivering both improved thickness tolerance and safety characteristics in case of window failure. These advances in entrance window technology prove essential for detecting fluorescence from light elements including sodium and magnesium, expanding the analytical capabilities of synchrotron beamlines.

Efficient detection at the opposite end of the energy spectrum presents a complementary challenge that requires a different approach. High-energy detection benefits primarily from increased SDD thickness. Standard 450 µm thick SDDs provide approximately 35% quantum efficiency at 20 keV, which proves insufficient for many demanding applications. Thicker SDDs, now commercially available in 1 mm or 2 mm configurations, substantially enhance performance in this energy regime. The 2 mm version achieves 88% quantum efficiency at 20 keV (Falkenberg *et al.*, 2020 ▸), representing more than a twofold improvement over standard thickness detectors. Although alternative semiconductor detectors including CdTe and HPGe offer detection efficiencies approaching 100% at 20 keV (Amptek Inc., 2024 ▸; ORTEC, 2024 ▸), these alternatives compromise the inherent performance advantages of SDDs through inferior energy resolution (Redus *et al.*, 2009 ▸). HPGe detectors additionally require cryogenic operation, introducing unwanted system complexity and operational constraints.

This paper presents ASCANIO (Annular SDD Configuration for Advanced Nano Imaging and Observation), a novel SDD-based X-ray spectrometer specifically designed for X-ray fluorescence microscopy (XFM) applications at high-brilliance synchrotron radiation facilities with low-fluorescence samples. The key design features implemented to address these challenges include high geometric efficiency through a total collimated active area of 324 mm^2^, high solid-angle coverage (1 steradian at 8 mm working distance), multi-element SDD arrays comprising 16 channels arranged in a tilted configuration to enhance uniform absorption at 10 mm working distance, and fast readout electronics. The following sections present detailed information regarding the conceptual design, performance characterization and commissioning results obtained on the P06 beamline (DESY, Germany).

## 2. ASCANIO motivations and working principle

XFM at synchrotron facilities plays a crucial role in performing elemental mapping, thanks to the high-brightness X-ray beam that can be focused in very small spots (down to a few tens of nanometres) (Schroer *et al.*, 2010 ▸; da Silva *et al.*, 2017 ▸). XFM evaluates the elemental composition and distribution of a sample by iterating measurements in a defined spatial grid, generating an overall image of the sample. A minimum dwell time per measurement is needed to collect enough photons and, depending on the size of the area of interest and the required spatial resolution, the time can range up to several hours. For example, with 1500 × 1500 pixels and a dwell time of 20 ms, the experiment will take around 12.5 h. The dwell time shows a lower bound limited by the fluorescence of the sample and by the solid angle covered by the detector, while the synchrotron beam intensity may need to be reduced because of limited detector output count-rate capability. These fundamental limitations constitute the primary motivation for developing ASCANIO: a novel spectrometer implementing a geometric architecture designed to maximize solid-angle coverage and count-rate uniformity featuring a total of 16 SDDs equipped with a fast low-noise electronic readout chain. ASCANIO has been developed at Politecnico di Milano for PETRA III (DESY) following the P06 beamline proposal.

The core design features of ASCANIO regard the geometric optimization. The system incorporates a 0.95 mm central aperture enabling backscattering geometry operation, an approach previously demonstrated by other detection systems (Siddons *et al.*, 2014 ▸; Rumancev *et al.*, 2020 ▸). This configuration substantially reduces the sample-to-detector distance, enabling significantly higher solid-angle coverage compared with lateral 90° detector arrangements. Additionally, the SDD modules are positioned in a tilted configuration, ensuring more uniform fluorescent light distribution among individual pixels (Section 3.1).

The ASCANIO detection unit comprises four independent four-channel modules assembled on a custom-fabricated aluminium support with a 20° tilt angle. This angle can be chosen to optimize collection and uniformity at a specific distance from the sample and must be evaluated in the design phase in collaboration with the final user.

The silicon thickness for the SDDs was selected at 1 mm to enhance absorption efficiency at energies up to 20 keV, as presented in Table 1 ▸ (BrukerNanoGmbH, 2025 ▸).

**Figure 1:**
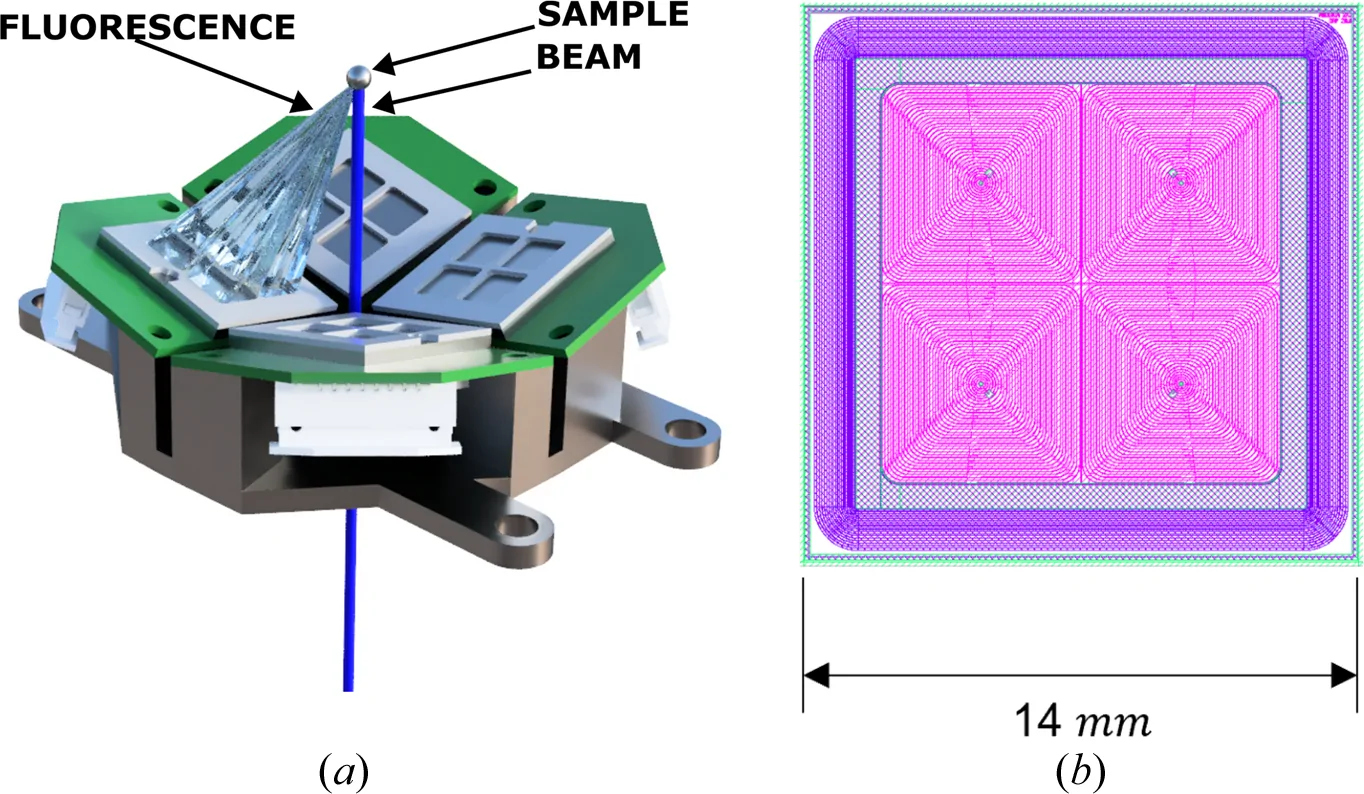
ASCANIO working principle and SDD layout. (*a*) Working principle of ASCANIO: the X-ray beam (highlighted in blue) crosses the detector head, hitting the sample. The emitted fluorescent light is then collected by four SDD modules, which are tilted and feature four channels each. (*b*) The SDD array has a squared geometry of 14 mm on a side and 1 mm thickness; each channel has a 5 mm × 5 mm active area.

The SDD arrays are provided by Fondazione Bruno Kessler (FBK). As shown in Fig. 1 ▸(*b*) ▸, the SDD geometry consists of a 14 mm × 14 mm square array, with each detector element having a 5 mm × 5 mm active area. On top of each SDD array, a 0.5 mm thick molybdenum collimator is placed to mitigate the effect of charge sharing between adjacent pixels, thus preserving the peak-to-background (PTB) ratio, determined by comparing the Mn *K*α peak height with average counts between 900 eV and 1100 eV. The collimator shields 0.25 mm from each side of the active pixel, reducing the active area of each detector to 4.5 mm × 4.5 mm. The four independent modules are arranged in an annular configuration around a hole where the X-ray beam will pass through. To understand better the working principle of ASCANIO, the reader is referred to Fig. 1 ▸(*a*) ▸. The entire system is encapsulated in a vacuum chamber made of aluminium to prevent ice formation in electronic components that are cooled to approximately −42°C, such as the sensor assembly. The X-ray beam crosses all the framework of the detector: lower window (black Kapton), water block, Peltier cells, aluminium support (which holds the SDDs and their collimators) and upper window (black Kapton). Afterwards, the beam hits the sample and the resulting fluorescent light is emitted and collected by the SDDs. Before reaching the upper window, the beam passes through a molybdenum needle (characterized by a 0.95 mm internal diameter and 1.5 mm external diameter), whose function is to absorb the scattered photons from the upper window and avoid them being captured by the sensors.

## 3. Instrument design

### 3.1. Solid angle and geometry analysis

Solid-angle maximization constitutes a critical design consideration for ASCANIO, as the fluorescent flux incident on the SDDs directly relates to the solid angles subtended between the sample and the SDD active areas. The SDD arrangement has been designed to optimize the detector solid angle while ensuring uniform fluorescent light distribution across all 16 channels (Ticchi *et al.*, 2022 ▸).

Uniformity is quantified using the max/min ratio (MMR), calculated from the solid angles subtended by the pixels positioned closest to (Ω_Max_) and furthest from (Ω_Min_) the central axis,
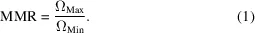
For each sample distance, a specific tilt angle exists where the MMR approaches unity. However, the mechanical structure imposes constraints due to the internal distance between the entrance window and the detector modules, which depends on the tilt angle.

Consequently, optimization at very short distances is not feasible, necessitating a compromise between the MMR and maximum count-rate capability. The final tilt angle selection must consider the experimental requirements and sample characteristics. For low-fluorescence-yield samples, shallower angles enable higher solid-angle coverage, whereas for high-fluorescence-yield samples, steeper angles provide improved count-rate distribution among channels, albeit with reduced overall solid-angle coverage. Notably, the solid-angle reduction resulting from the tilted configuration compared with a flat configuration at a fixed distance is minimal. Calculations demonstrate that at a 10 mm sample distance, a 20° tilt produces a less than 10% solid-angle reduction with respect to a flat configuration of detectors, while improving the MMR from 2.3 to 1.1.

**Figure 2:**
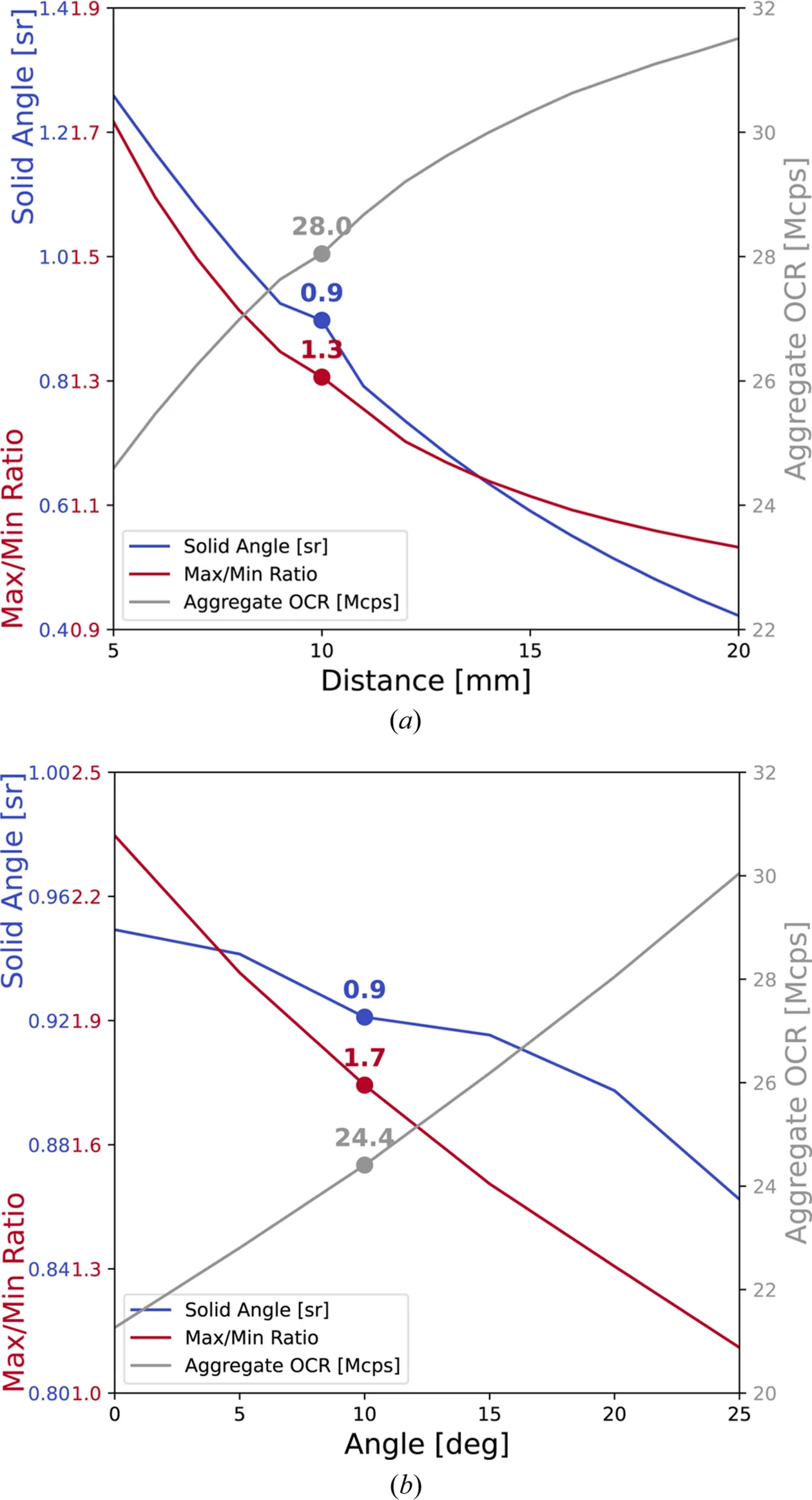
Solid angle, MMR and aggregate maximum output count rate (OCR) plotted against distance from the window and module tilt angle. (*a*) Detector figures of merit as a function of distance from the window (20° tilt angle). A maximum OCR of 2 Mcps has been considered for the units closer to the center. (*b*) Detector figures of merit as a function of module tilt angle (10 mm distance from the window).

Fig. 2 ▸(*a*) ▸ shows the dependence of the solid angle, MMR and maximum theoretical output count rate (OCR) for different distances from the instrument window, with a fixed tilt angle of 20°. The aggregate OCR has been estimated considering a maximum OCR of 2 Mcps for the channels closer to the center, a value achievable with modern DPPs (Pedretti *et al.*, 2025 ▸). Fig. 2 ▸(*b*) ▸ shows the dependence of the solid angle, MMR and maximum theoretical OCR on the module tilt angle, with a fixed 10 mm distance from the instrument window. Due to the window structure, the effective distance of the modules from the samples increases with the tilt angle, greatly impacting the effective solid angle covered by the instrument. The OCR, however, depends only on the MMR and increases with the angle. The selected values are highlighted in the figure. For the P06 beamline application, the tilt angle was selected to achieve an optimal compromise between working distance and uniformity. The design is optimized for a 20° angle and 10 mm distance from the window, a distance considered safe enough not to risk collisions on the detectors while hanging/translating the sample, enabling tomographic XFM experiments. While the angle is fixed in the design, the distance from the sample can be easily modified on the beamline.

### 3.2. Electronics

**Figure 3:**
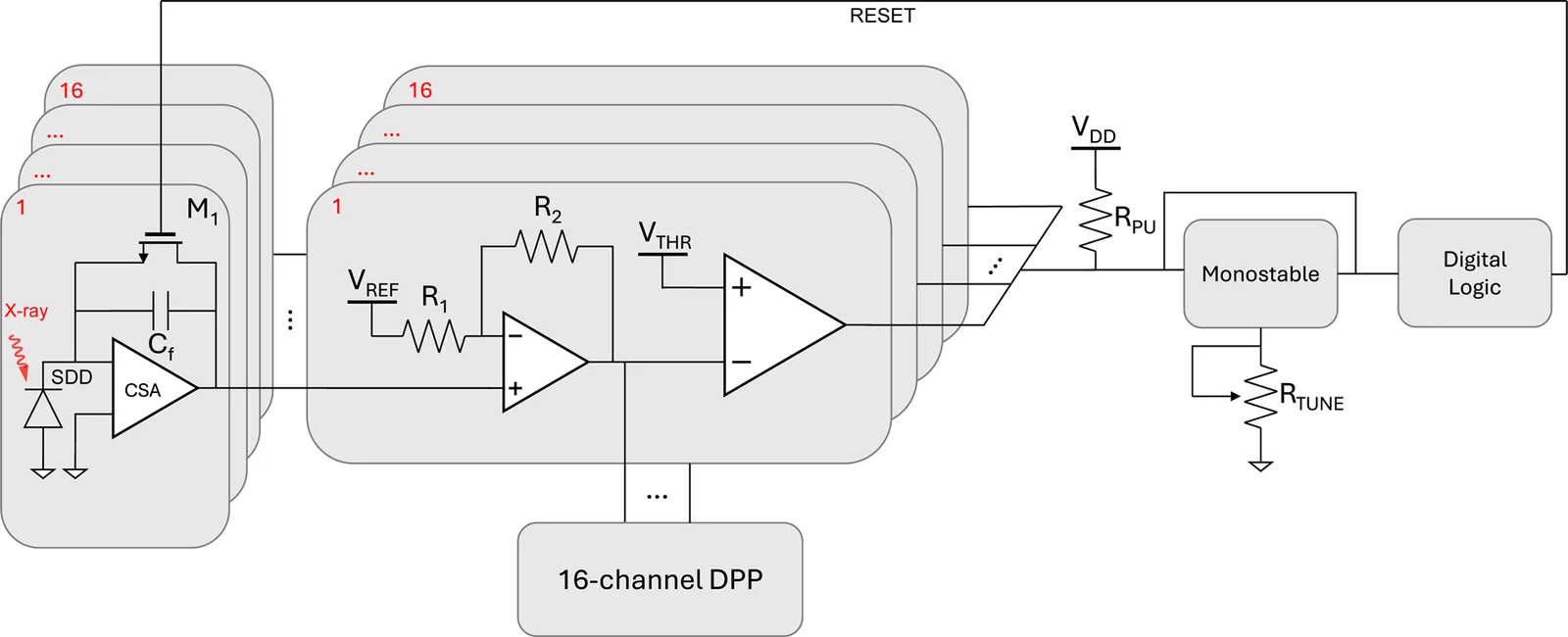
Simplified schematic diagram of the electronics of the system.

Fig. 3 ▸ presents a schematic view of the ASCANIO spectrometer electronics readout chain. Photo-generated charge is integrated by the charge sensitive amplifier (CSA) and converted to a voltage signal. The selected CSA is a four-channel CUBE, a low-noise charge preamplifier developed at Politecnico di Milano in 2011 and optimized for radiation detector applications (Bombelli *et al.*, 2012 ▸; Bombelli *et al.*, 2011 ▸). An adjustable offset (*V*_REF_) and fixed-gain analog stage match the signal voltage swing to the digital pulse processor (DPP) dynamic range. For this purpose, a low-noise operational amplifier in non-inverting configuration is employed for each channel. Reset generation for the CUBE preamplifiers is performed by dedicated circuitry, also shown in Fig. 3 ▸. The comparator output stage utilizes an open-drain configuration with a single pull-up resistor (*R*_PU_); when any of the 16 channels exceeds the adjustable threshold (*V*_THR_), the comparator output switches and triggers a monostable circuit. The monostable circuit generates a pulse with a duration adjustable using a tunable resistor (*R*_TUNE_), setting the reset time. The RESET signal is provided to the CUBE preamplifier using minimal logic to match the input requirements.

Each module comprises a printed circuit board (PCB) hosting a 1 mm thick four-channel SDD monolithic array directly wire-bonded to a four-channel CSA. The array is collimated with a molybdenum collimator fixed directly to the sensor surface. Special care was taken in the choice of PCB material; by using halogen-free FR4 instead of the standard one we can minimize unwanted lines in the spectrum due to board fluorescence.

The wire bonding process is critical for achieving optimal performance. The compact CSA footprint enables close proximity to the sensor, maintaining a minimal bonding wire length and thereby reducing parasitic capacitance. By wedge-bonding the anodes directly to the four-channel CSA, the length of the connections is limited to a few millimetres.

**Figure 4:**
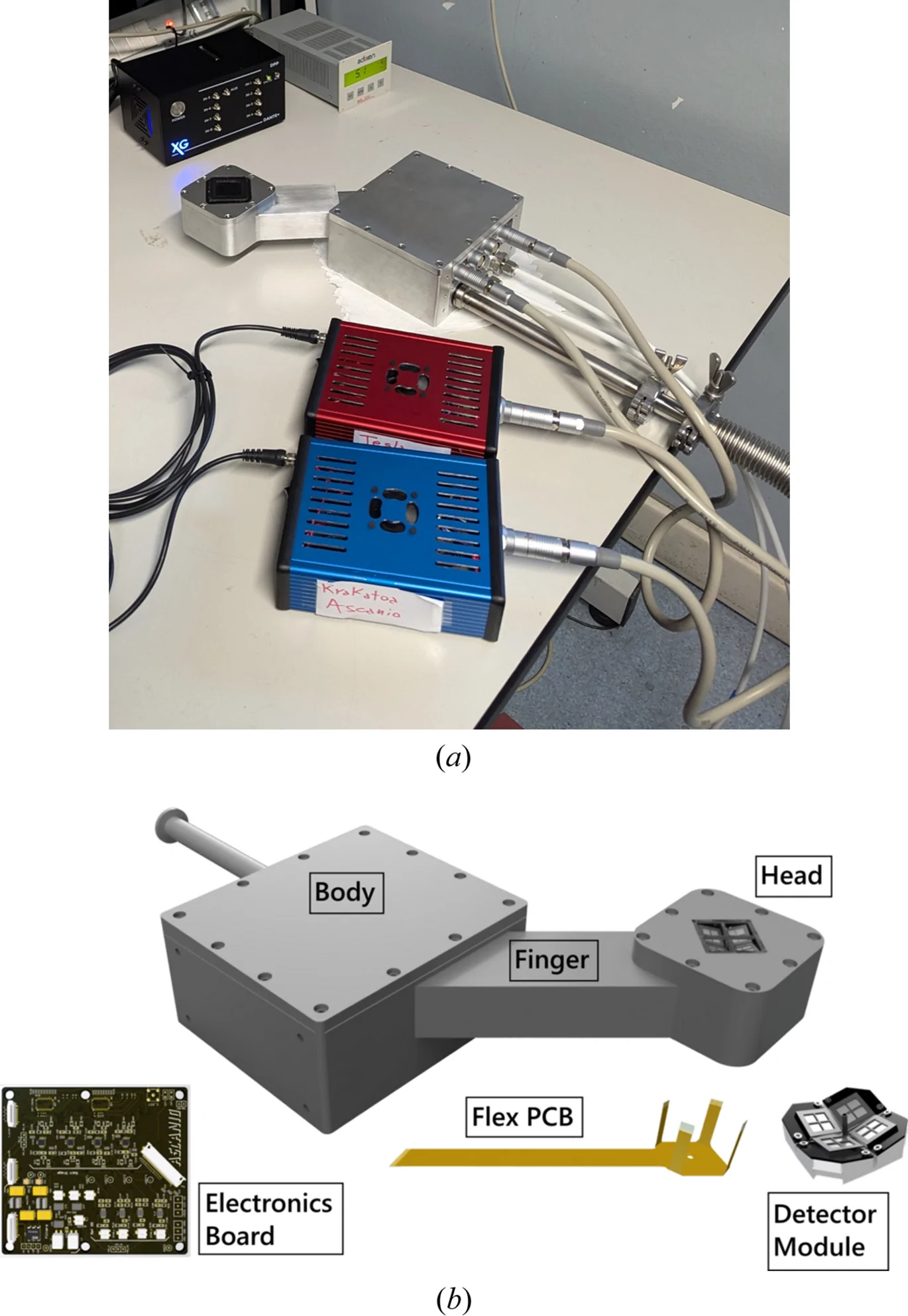
ASCANIO instrument assembly and main components. (*a*) Laboratory setup of ASCANIO together with the external units. The figure also shows all the electrical connections, a pair of tubes for liquid cooling and the vacuum connections. (*b*) Simplified schematic diagram of the instrument, highlighting the mechanical structure and main components.

The modules include only the SDD and CUBE preamplifier, with direct connection to the supporting electronics housed in the main body. Connection is achieved using a Kapton-based flexible PCB that wraps around the sensing head rear surface, connecting the four modules to the readout board. Board positioning is presented in Fig. 4 ▸(*b*) ▸.

The readout board, housed in the main body, performs several functions. First, it processes the CSA signal and performs signal conditioning to adapt it to the DPP inputs. Second, it generates the tunable reset signal required by the CSA, accounting for all channels and generating a single reset signal for all modules. The system also supports reset suppression in the eventuality that some channels are damaged during the detector lifetime. Third, it generates all power supplies and bias voltages necessary for proper sensor operation.

External connections are provided through Fisher hermetic circular connectors, connected to the readout board via flexible flat cables. On the atmospheric side, the instrument connects to two external units. TESLA [red unit in Fig. 4 ▸(*a*) ▸] provides the instrument power supply, generating all required voltages while continuously measuring and monitoring all voltages and currents for fault recognition. KRAKATOA [blue unit in Fig. 4 ▸(*a*) ▸] manages thermal control, driving the thermoelectric cooler and monitoring system temperatures by reading Pt100 temperature sensors. These external units are controlled via a USB interface from a computer (Utica *et al.*, 2021 ▸).

Additionally, two Fisher connectors accommodate eight coaxial cables each for DPP connection and instrument readout. These cables are terminated according to beamline DPP specifications.

### 3.3. Mechanics

ASCANIO is designed for use on atmospheric pressure end stations, while the detector itself operates under vacuum to optimize cooling performance. The level of vacuum needed for ASCANIO (around 10^−5^ mbar) is far from the UHV range (less than 10^−8^ mbar) and the instrument does not share the vacuum with any critical equipment on the beamline. Nevertheless, several optimizations have been implemented to reduce the pumping effort. The overall housing is fabricated from aluminium with vacuum-tight welding between components. The internal volume has been minimized; although maintaining only the sensing head under vacuum would be optimal, implementing internal vacuum feedthroughs between separate bodies presents substantial challenges regarding both geometric constraints and electrical performance. Consequently, a single connected volume solution was implemented. Material selection prioritized vacuum-compatible options, utilizing plastics such as polytetrafluoroethylene (PTFE) and polyimides wherever feasible.

Fig. 4 ▸(*a*) ▸ presents the final ASCANIO instrument assembly, together with the supporting external electronics. The main instrument comprises two main bodies housing the detection unit and supporting electronics, respectively. These bodies are connected via a welded custom tube that defines their relative positions and overall instrument dimensions.

**Figure 5:**
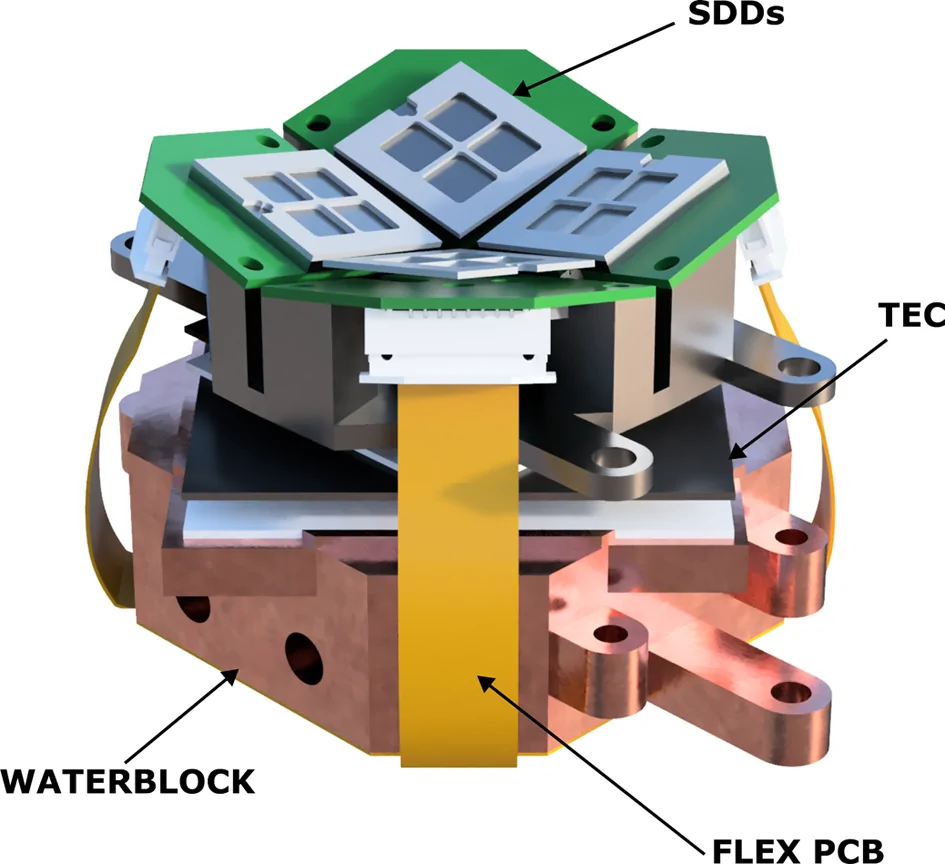
Three-dimensional render of ASCANIO sensing head internals, with highlights on the monolithic modules (SDDs), the thermoelectric coolers (TEC), the 3D-printed heat exchanger (WATERBLOCK) and the flexible PCB for connection with the readout board (FLEX PCB).

The sensing head consists of a square aluminium enclosure slightly larger than the detector unit. The internal components are arranged in a layered stack configuration that can be seen in Fig. 5 ▸. At the base of the stack, a liquid heat exchanger provides heat removal for the hot side of the thermoelectric coolers. This heat exchanger is a custom design since it needs several unique features, such as anchoring points for the stack and a central aperture. The component was manufactured in aluminium, using metal 3D printing, to optimize thermal conductivity. Two Peltier modules positioned on top of the heat exchanger provide sub-zero cooling to the sensing head and sensors. System temperatures on both hot and cold sides of the thermoelectric coolers are continuously monitored using two Pt100 temperature sensors and can be used to recognize good operating conditions or even faults in the cooling system.

The stack assembly is secured with PTFE screws to minimize thermal conduction to the frame, avoiding adhesives or permanent bonding. The aluminium enclosure is sealed with two hermetic caps hosting X-ray windows on both front and rear surfaces. The head connects to the main body through a square tube fixed with vacuum-tight welds. The system structure is clearly illustrated in Fig. 4 ▸(*b*) ▸. The relative positioning of the head and main body is critical and was designed specifically for the requirements of the receiving beamline. The connecting tube provides mechanical support for the head, enables vacuum sharing between bodies and serves as a conduit for electrical connections and hydraulic lines.

The main body serves as the primary mechanical mounting interface within the beamline, with mounting points designed in collaboration with the P06 beamline team. This body houses the readout board and provides hermetic connectors for all necessary connections, including output signals for external pulse processors, power supply connections, and thermal management and monitoring interfaces. Additionally, the main body provides a KF16 vacuum fitting for the complete instrument and hydraulic fittings for the liquid cooling loop.

Vacuum performance testing was conducted using a benchtop turbomolecular pump via a 25 mm internal diameter flexible tube approximately 1 m long, achieving a vacuum level of approximately 5.1 × 10^−6^ mbar measured at the instrument fitting.

## 4. Laboratory characterization

Prior to detector commissioning on P06, the ASCANIO instrument was assembled in its final enclosure and comprehensively tested at the Politecnico di Milano laboratory using a ^55^Fe radioactive source.

The vacuum performances were checked, and the instrument can maintain a good level of vacuum, reaching down to around 10^−6^ mbar. Thermal performances were verified and monitored across several days to identify possible failures in the cooling components: the liquid cooling chiller was set to 15°C to prevent condensation on the tubing, and the 3D-printed waterblock maintained the hot side of the Peltier below 17°C. The thermoelectric cooler achieved approximately −42°C with the instrument active.

Electrical testing confirmed that all 16 channels are operational, indicating that the sensor did not sustain damage during the handling, bonding and mounting process. At the operational temperature of around −42°C, with the instrument in full operational conditions, the instrument shows 16 ramps, with reset intervals of approximately 85 ms.

**Figure 6:**
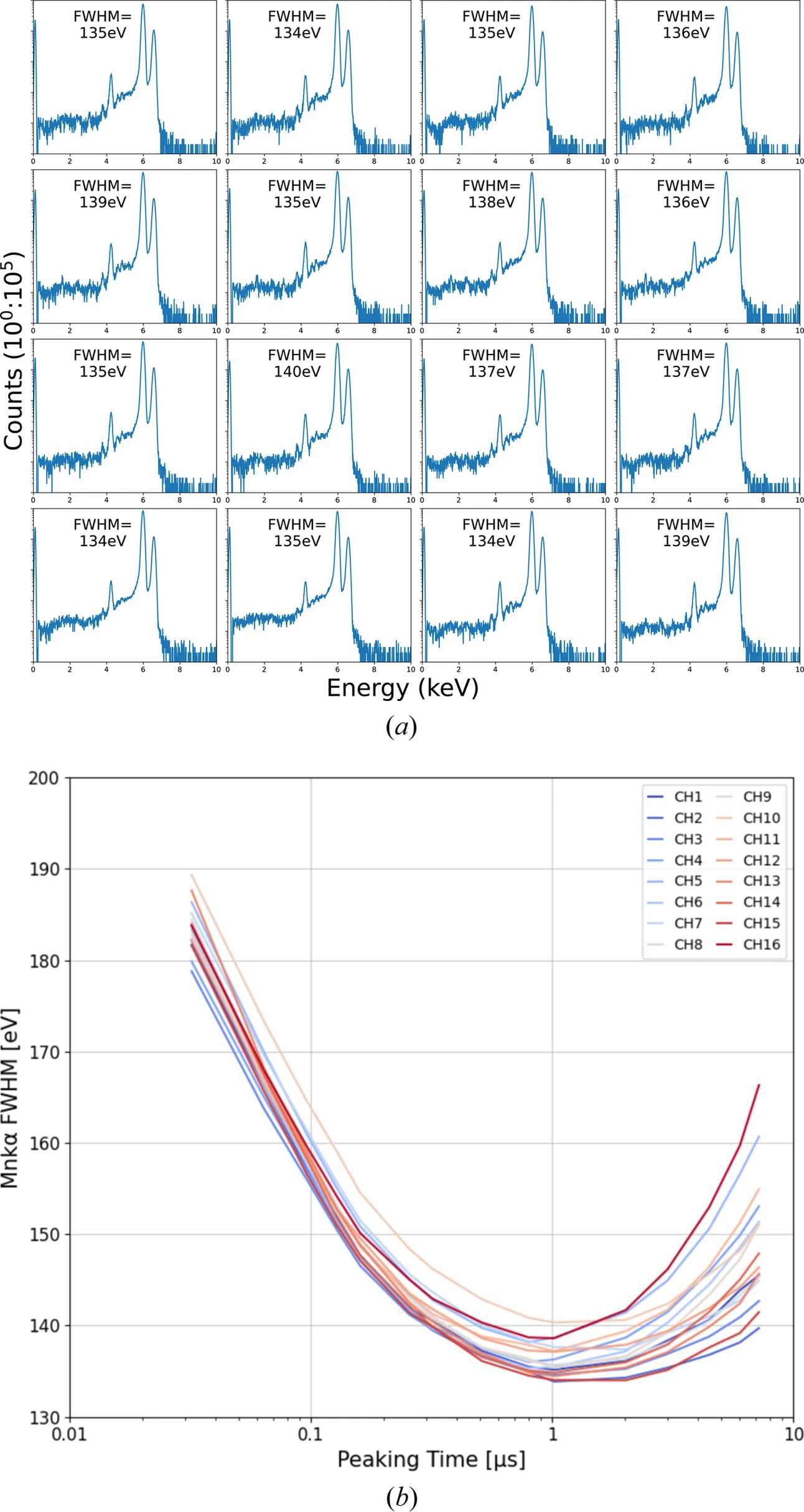
(*a*) Spectra measured on each channel for 30 s at 45 kcps per channel, highlighting the energy resolution at 5.9 keV. (*b*) Energy resolution at 5.9 keV at different peaking times measured at 45 kcps per channel.

Preliminary measurements were performed using DANTE (Iguaz *et al.*, 2023 ▸), a digital pulse processor (DPP) from XGLab s.r.l. (Milan, Italy) based on trapezoidal pulse filtering. Count rates were varied by adjusting the source distance, and consequently the incident radiation intensity, up to a maximum of approximately 400 kcps per channel. Fig. 6 ▸(*a*) ▸ presents representative spectra acquired at an optimal peaking time of 800 ns.

Fig. 6 ▸(*b*) ▸ presents the performance of the 16 SDDs at various peaking times. At the minimum peaking times of 32 ns all the channels achieve a resolution below 190 eV FWHM at the Mn *K*α line (5.9 keV). At the optimal peaking time of 1 µs, only one channel exceeds 140 eV resolution, with the best-performing channels achieving 134 eV FWHM. Tests were performed varying the input count rate (ICR), and the results at 45 kcps and 400 kcps are summarized in Table 2 ▸.

**Figure 7:**
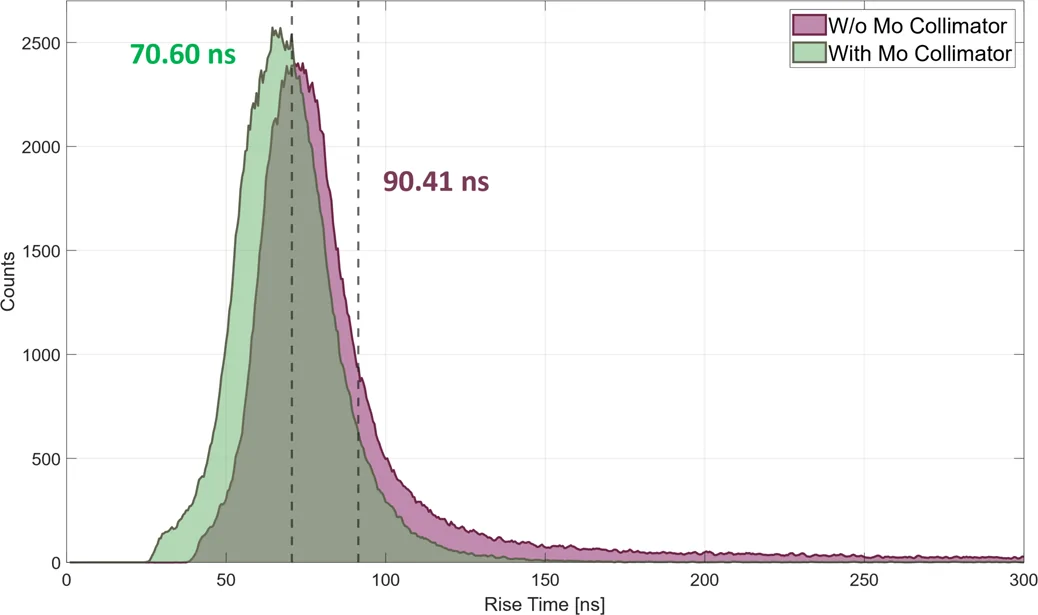
Rise-time distribution of the system, comparing the effect of collimator presence. The average values of the curves are highlighted in the graph.

Rise-time measurements demonstrate the collimator’s effectiveness in shielding charge collection at edges and corners, where pulses exhibit slower behavior. The collimator effectively reduces the average pulse rise time from 90 ns to 70 ns. This reduction is a clear indication of the mitigation of charge-sharing effects. These happen when a photon is collected at a boundary, splitting the generated charge between two pixels. This causes invalid lower-energy counts, ultimately worsening both the resolution and PTB ratio. The event rise-time distribution is presented in Fig. 7 ▸, showing a direct comparison between collimated and uncollimated configurations.

The instrument, measured at an OCR of 40 kcps, achieved an average PTB ratio of 8600 across all channels, with the best-performing channels achieving a PTB ratio of 11800.

## 5. Beamline characterization

The instrument was commissioned in June 2025 on the P06 beamline at PETRA III (DESY). P06 is a hard X-ray micro/nanoprobe beamline, capable of various X-ray techniques including X-ray fluorescence (XRF), X-ray absorption spectroscopy (XAS) and X-ray diffraction (XRD). The beamline comprises two main experimental hutches utilizing separate X-ray optics systems to provide different focal spot sizes according to specific experimental requirements (Schroer *et al.*, 2010 ▸). The microprobe hutch emphasizes versatile X-ray microscopy experiments where dwell-time reduction is critical and constitutes the primary motivation for ASCANIO development.

**Figure 8:**
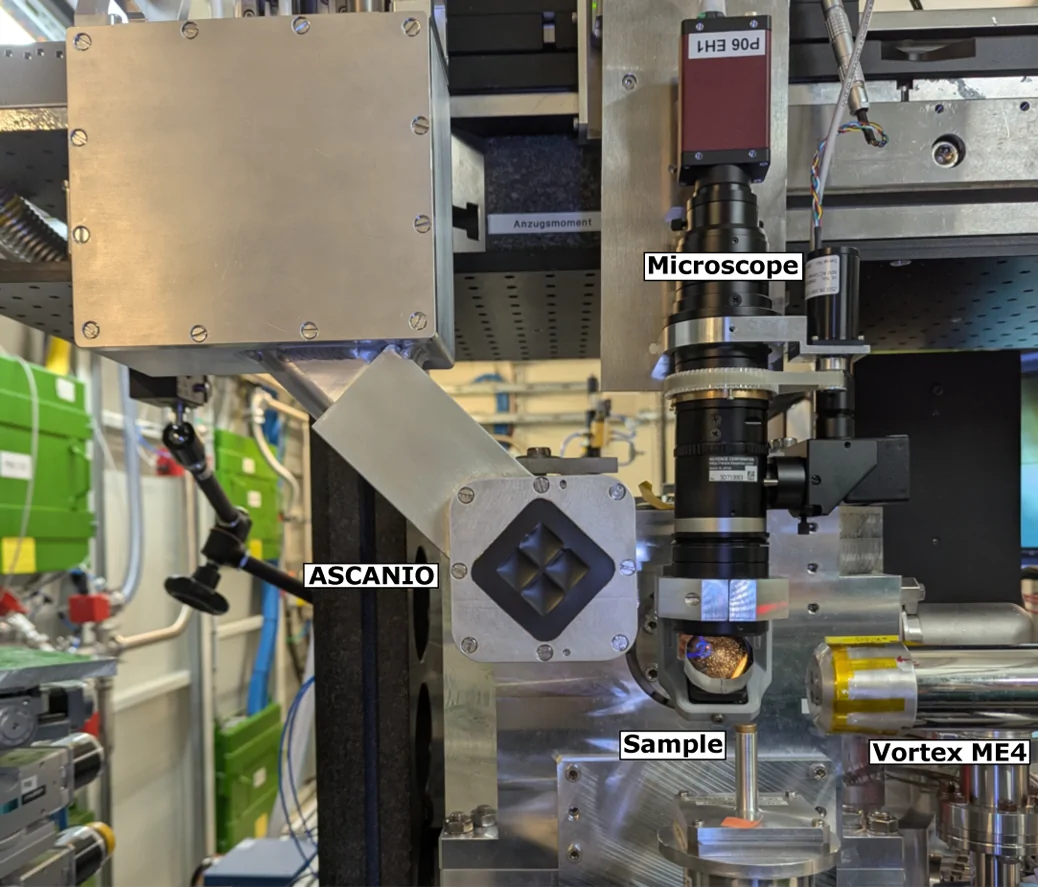
The instrument mounted on the P06 beamline, showing ASCANIO itself, the optical microscope, a lateral four-channel spectrometer and the sample holder.

Instrument commissioning was performed in collaboration with beamline staff in the experimental hutch. All mechanical and mounting features required by end users were analyzed during the design process, enabling rapid deployment. Fig. 8 ▸ presents the instrument installed on the beamline. ASCANIO is mounted on a linear rail for rapid and automatic centering, enabling quick exchange with other beamline instruments. All required connections are positioned on the instrument top surface to maintain cables and tubing away from the working space.

The P06 acquisition system utilizes commercial Xspress3-mini (Quantum Detectors, 2025 ▸) pulse processors with a total of 40 acquisition channels, enabling simultaneous operation of ASCANIO, ARDESIA-16 (Utica *et al.*, 2021 ▸) and a Vortex-ME4 (Hitachi High-Tech America Inc., 2025 ▸) detector. Instrument calibration was performed *in situ* using both the synchrotron beam with a manganese foil target and a radioactive ^55^Fe source.

**Figure 9:**
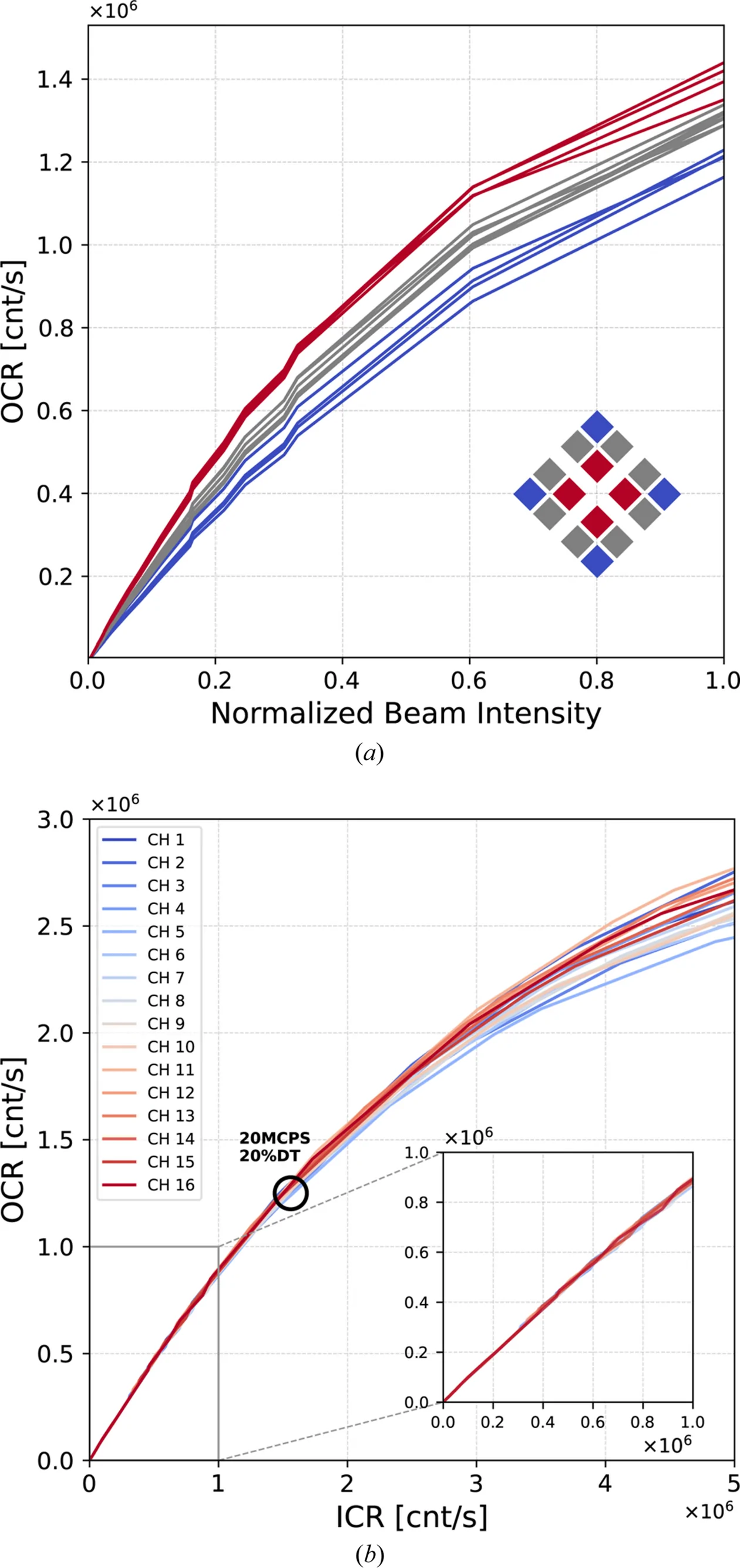
(*a*) Plot of OCR against beam intensity, highlighting the different solid-angle coverage of the channels. (*b*) Plot of OCR against ICR, showcasing the almost-linear region at lower ICR.

During the commissioning process several key performances were verified, starting with the centering of the detector. By translating the detector across the beam, it is possible to find the edges of the central opening. This reaches the maximum only if the internal mechanical structure of the sensing head has sufficient alignment, since several components must maintain a passage for the incoming beam. Fig. 9 ▸(*a*) ▸ shows the OCR as a function of the beam intensity. Here, three distinct groups of channels are clearly visible and color coded. This stems from the geometric positioning of the modules, where the channels can be grouped in three coronas depending on the distance from the center. After confirming basic instrument functionality and mechanical alignment, the test continued with the verification of high count-rate capabilities. By varying the beam intensity *I*_0_, ICR up to 10 Mcps per channel was achieved.

Fig. 9 ▸(*b*) ▸ reports the relationship between OCR and ICR measured by the Xspress3 for all 16 channels. An ideal system would show a perfectly linear relationship, but with increasing ICR the performances of both the detector and the supporting electronics are impacted, introducing dead time. Considering OCR/ICR > 0.8 as a reference acceptability limit, the detector can reach up to 20 Mcps aggregate OCR. At 1 Mcps per channel, the detector achieves between 11.7% and 15.4% of dead time across all 16 channels, averaging around 13.5%.

Testing continued with the verification of the ‘tilted sensor’ structure. The tilt angle of ASCANIO is fixed at the design stage and cannot be easily changed in a second phase without the need for total disassembly of the sensing head, so the geometric improvements were verified by varying the detector distance from the sample. Since the detector design is optimized for isotropic emission from samples, verification was performed considering only sample fluorescence (region of interest on the Mn *K*α peak), excluding elastic and inelastic scattering peaks.

**Figure 10:**
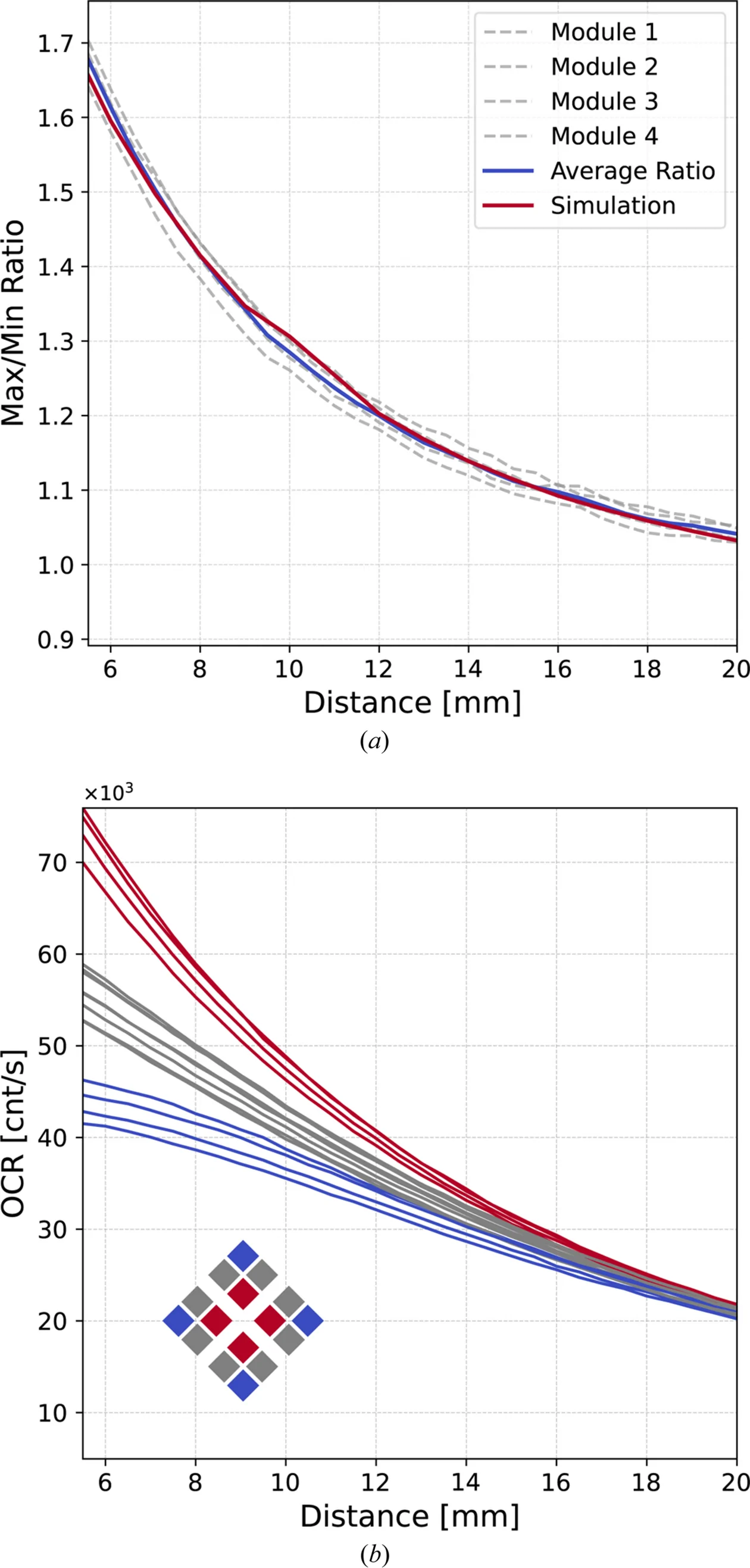
(*a*) Measurement of the instrument MMR versus sample distance, compared with the simulated data. (*b*) Plot of the OCR of the various channels versus sample distance.

Fig. 10 ▸(*a*) ▸ presents the obtained results, highlighting the overall instrument MMR, calculated by averaging the module results, and the theoretical MMR from simulation data, showing good accordance with the calculated models. Fig. 10 ▸(*b*) ▸ highlights the tradeoff that exists between the MMR and the overall solid angle. Depending on the intensity of the sample fluorescence and the experimental requirements, the ASCANIO position can be selected accordingly. In a low-fluorescence-yield sample, a closer distance can maximize the solid angle, increasing the OCR, while for a sample with higher fluorescence yield, where the count rate per channel is likely to be limited by the electronics, increasing the distance can provide a more uniform irradiation of the channels.

## 6. Experimental results with synchrotron beam

In the months following commissioning, the ASCANIO system was thoroughly tested on beamline P06 and applied in user experiments. We report here one example that showcases ASCANIO’s performance and highlights the advantages that this system can provide to the beamline.

A 10 µm thick section of *post mortem* brain tissue (87-year-old female donor; cause of death: cardiac failure; *post mortem* interval before fixation: 24 h) containing the substantia nigra region was used for quantitative elemental mapping using XRF and an ASCANIO detector. Full details regarding donor information and sample preparation can be found in the report by Friedrich *et al.* (2021 ▸; Case 3, Fig. 2). The section contained neuromelanin-pigmented dopaminergic neurons, which are known to accumulate iron. The section was stained for tyrosine hydroxylase using Ni-enhanced immuno­histochemistry to visualize dopaminergic neuron bodies, based on enhanced nickel concentration.

**Figure 11:**
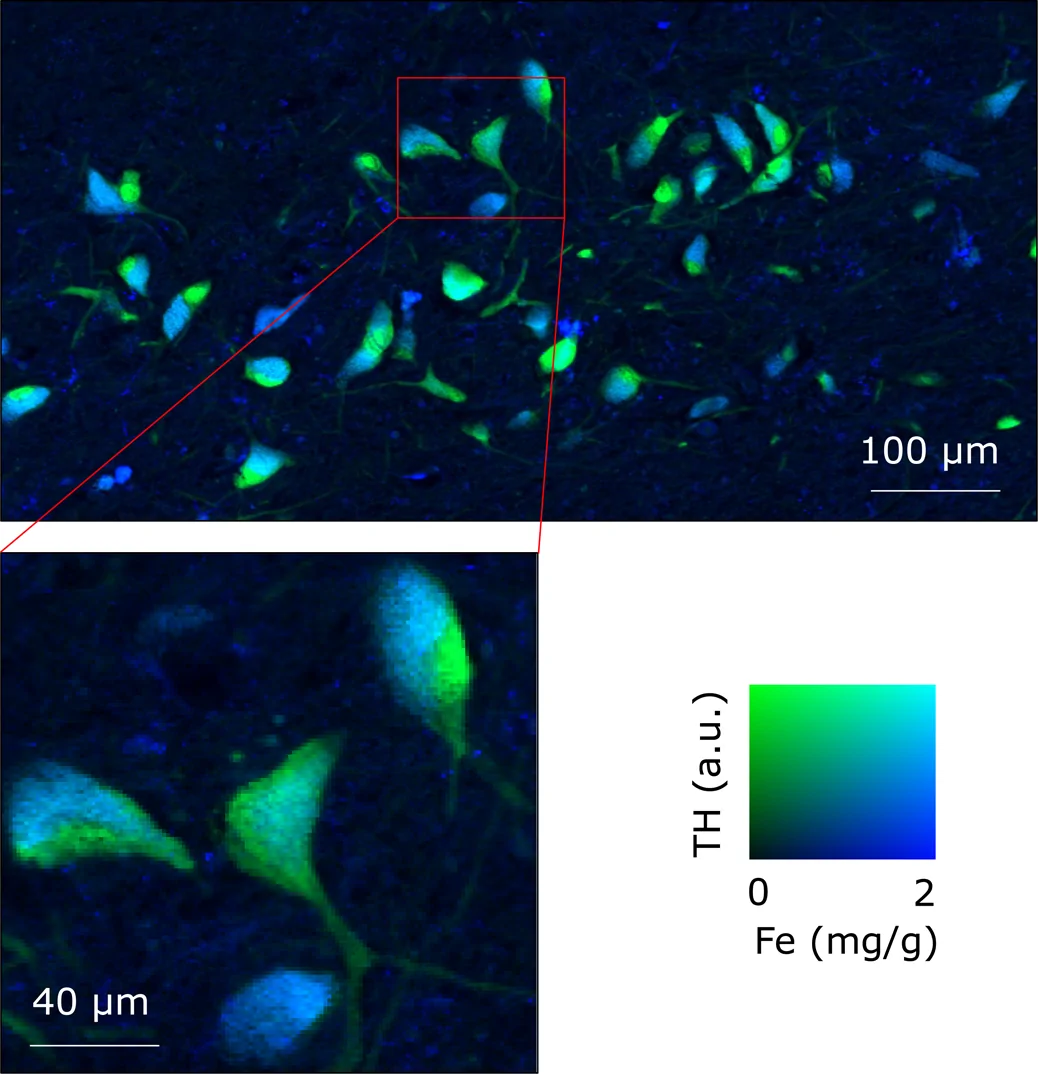
Quantitative maps of iron distribution in a human brain section from the substantia nigra, obtained using the ASCANIO detector. The overview scan at the top, with a section enlarged below, shows a region encompassing nigrosome 1, highlighting elevated iron levels in tyrosine hydroxyl­ase-positive dopaminergic neurons (Friedrich *et al.*, 2021 ▸; Brammerloh *et al.*, 2021 ▸; Brammerloh *et al.*, 2024 ▸). Hotspots of increased iron concentration correspond to the locations of iron-rich neuromelanin-pigmented neurons. The high sensitivity of the detector enables imaging of macroscopic areas at microscopic resolution within reasonable scan times, allowing for the mapping of iron concentrations both in individual cells and across large neuronal ensembles.

Quantitative maps of iron and nickel in the substantia nigra region are shown in Fig. 11 ▸. The overall scan covers an 800 µm wide × 400 µm high section of tissue with a 1 µm resolution, with 7 ms dwell time, 4 × 10^10^ photons s^−1^ in focus and 13 keV excitation energy. The group of dopaminergic neurons exhibits elevated iron concentrations, consistent with previous findings (Friedrich *et al.*, 2021 ▸). The high sensitivity of the ASCANIO detector enabled precise mapping of cellular iron concentrations with micrometre resolution across large areas containing multiple cells, thus providing both cellular specificity and statistical robustness over large neuronal ensembles within a short scanning time (40 min).

The ASCANIO system, while not strictly necessary for these analyses, could significantly lower the required dwell time to obtain the same statistics as the previous system, enabling mapping of larger sized samples.

## 7. Conclusions

The first ASCANIO spectrometer has been completed and commissioned. The instrument underwent preliminary testing in the laboratory, where vacuum and cooling performances were verified, achieving around −42°C with an internal pressure of 5.1 × 10^−6^ mbar. The 16 channels were tested with a ^55^Fe source, where the instrument achieved resolutions as low as 134 eV FWHM at the Mn *K*α peak. ASCANIO was later installed on beamline P06 at PETRA III (DESY), where it was thoroughly tested. The geometric enhancements confirmed the uniformity improvements related to pixel solid-angle coverage, reaching a minimum MMR below 1.3. The detector demonstrated very good high rate performances, achieving a 20 Mcps cumulative OCR with a dead time of 20%. The spectrometer is now in use on the beamline for XFM experiments, where the improved solid angle enables a shorter dwell time, reducing the total experiment duration.

## Figures and Tables

**Table 1:** Comparison of SDD absorption efficiency for different thicknesses of 450 µm and 1 mm

Energy (keV)	450 µm SDD (%)	1 mm SDD (%)
10.0	97.37	99.97
20.0	37.29	64.55
30.0	13.78	28.07
40.0	6.96	14.80

**Table 2:** Performances of the detector measuring the energy resolution at 5.9 keV Best, average and worst pixel performances are shown for both the fastest setting available (32 ns) and for the peaking time that achieves the best energy resolution (reported in parentheses in the ‘opt.PT’ column), which can have some differences depending on the pixel and the count rate.

	45 kcps		400 kcps	
	32 ns	opt.PT	32 ns	opt.PT
Minimum	179 eV	134 eV (1 s)	180 eV	139 eV (800 ns)
Average	184 eV	136 eV (1 s)	186 eV	148 eV (512 ns)
Maximum	189 eV	140 eV (1 s)	191 eV	159 eV (256 ns)

## Data Availability

The data that support this study are available from the corresponding author upon request.
